# Low-Data Drug Design with Few-Shot Generative Domain Adaptation

**DOI:** 10.3390/bioengineering10091104

**Published:** 2023-09-21

**Authors:** Ke Liu, Yuqiang Han, Zhichen Gong, Hongxia Xu

**Affiliations:** 1 College of Computer Science and Technology, Zhejiang University, Hangzhou 310027, China; lk2017@zju.edu.cn; 2 ZJU-Hangzhou Global Scientific and Technological Innovation Center, Hangzhou 311200, China; zhichen.gong.18@ucl.ac.uk; 3 Department of Computer Science, University College London, London WC1E 6BT, UK; 4 Innovation Institute for Artificial Intelligence in Medicine, Zhejiang University, Hangzhou 310027, China

**Keywords:** drug design, domain adaptation, generative model

## Abstract

Developing new drugs for emerging diseases, such as COVID-19, is crucial for promoting public health. In recent years, the application of artificial intelligence (AI) has significantly advanced drug discovery pipelines. Generative models, such as generative adversarial networks (GANs), exhibit the potential for discovering novel drug molecules by relying on a vast number of training samples. However, for new diseases, only a few samples are typically available, posing a significant challenge to learning a generative model that produces both high-quality and diverse molecules under limited supervision. To address this low-data drug generation issue, we propose a novel molecule generative domain adaptation paradigm (Mol-GenDA), which transfers a pre-trained GAN on a large-scale drug molecule dataset to a new disease domain using only a few references. Specifically, we introduce a molecule adaptor into the GAN generator during the fine tuning, allowing the generator to reuse prior knowledge learned in pre-training to the greatest extent and maintain the quality and diversity of the generated molecules. Comprehensive downstream experiments demonstrate that Mol-GenDA can produce high-quality and diverse drug candidates. In summary, the proposed approach offers a promising solution to expedite drug discovery for new diseases, which could lead to the timely development of effective drugs to combat emerging outbreaks.

## 1. Introduction

Drug discovery and development are critical translational science activities that significantly contribute to human health and well-being [1]. However, drug discovery is a long-term, high-investment, and high-risk endeavor that traditionally relies on human expertise to design, synthesize, and test new drug molecules [2,3]. Traditional drug design methods can take an average of 6 to 12 years and cost billions of dollars to produce just one drug [4,5]. While only an estimated $10^{8}$ compounds have ever been synthesized, the theoretical number of feasible compounds ranges from $10^{23}$ to $10^{60}$ [6]. As a result, conventional discovery methods can only explore a limited amount and diversity of chemical space. Therefore, there is an urgent need to develop efficient methods for exploring chemical space to accelerate and improve the drug discovery process.

In recent years, deep learning technology has been utilized to expedite and enhance the drug discovery process [7,8,9]. Specifically, bioinformatics scientists have shown a keen interest in generative models due to their remarkable capacity to comprehend and explore the intrinsic properties of data [10,11]. Rather than relying on human expertise to design molecules, generative models employ recent advancements in deep learning to tackle the inverse molecular design problem: determining the set of molecules that will satisfy a desired set of properties [3]. Generative models can swiftly identify a wide range of molecules that are optimized for specific goals by mapping properties to structures. Recently, there has been a significant increase in the number and diversity of generative models employed in molecular design, such as variational autoencoders (VAEs) [12], generative adversarial networks (GANs) [13], and normalizing flow models [14]. In generative models, drug molecules are mostly represented as strings, such as SMILES (Simplified Molecular Input Line Entry System) [15] and SELFIES (Self-Referencing Embedded Strings) [16], or graphs [17]. For instance, VAEs have been utilized to generate SMILES strings and molecular graphs by approximately maximizing likelihood through variational inference techniques [18,19,20]. Similarly, GANs have also been adapted to generating molecules represented as sequences or graphs by formulating molecule generation as a minimax game [21,22,23]. Furthermore, normalizing flow models generate molecules by learning a series of invertible transformations between high-dimensional molecule data and a prior distribution [24,25,26].

Although generative models have made significant progress, their effectiveness primarily relies on the amount of training data, with larger sample sizes leading to greater accuracy. Unfortunately, acquiring labeled data for emerging diseases like COVID-19 can be challenging. The characteristics of effective drugs for such diseases are not yet established, and only a limited number of drugs are available to alleviate symptoms. As a result, training a generative model with adequate performance for low-data drug discovery is a daunting task due to the limited availability of labeled data. Few-shot generative domain adaptation has been introduced to address the challenge of limited data availability in GAN training [27,28,29]. Typically, a large-scale model is first trained in the source domain with a sufficient amount of data and then transferred to the target domain with only a few samples. Building on this idea, we propose a novel few-shot Molecule Generative Domain Adaptation paradigm (Mol-GenDA) for the low-data drug design. Specifically, we introduce a lightweight module called the molecule adaptor, which aids in adapting the generator to the target disease with the target molecule’s attributes. We first pre-train the GAN on a large-scale drug-like dataset, then freeze the parameters of the pre-trained generator and optimize only the molecule adaptor during fine tuning on the new disease dataset. This approach leverages the prior knowledge learned in the source domain to inherit the generation quality and diversity of the source model.

We have conducted extensive experiments to evaluate the proposed method’s ability to generate molecules with specific structures and desired properties in low-data drug design. The experiments showed that the proposed method can generate both simple structural features, such as halogen groups or aromatic rings, and more complex molecules with higher scores of desired properties, such as penalized logP and quantitative estimate of drug-likeness (QED score), among others. As part of our study, we have designed drugs that could be effective against COVID-19 and assessed the properties of various drug candidates.

## 2. Research Problem and Motivation

### 2.1. Research Problem

The aim of this study is to develop a generation method for the rapid design of effective drugs for emergent diseases. The main challenge in achieving this goal is training an effective generative model on only a few referenced drug molecules. Moreover, emerging diseases often require drugs with multiple desired properties, further complicating the generation process. Therefore, the generation method needs to address these challenges, namely few-shot reference drugs and multiple desired properties. The low-data drug design problem can be formalized as follows: If we only have a few known drug molecules $M_{r}$ that are partially effective in treating a specific disease, such as relieving certain symptoms, how can we train a generative model $f_{\theta }$ to design new drugs based on this information?

Generative models are expected to possess two key capabilities: (1) structure-constrained generation, which involves the ability to make simple structural modifications such as altering the presence of halogen groups or adjusting the number of aromatic rings; and (2) property-constrained generation, which enables the model to generate molecules with higher scores of desired properties. By leveraging these abilities, generative models can produce drug molecules that are more effective in treating emerging diseases or are easier to manufacture.

### 2.2. Limitation of Previous Methods

**VAEs.** Variational autoencoders (VAEs) are widely used in drug design and consist of an encoder and a decoder [12]. The encoder converts a molecule into a latent vector representation and maps it to a pre-defined distribution of valid molecule latent vectors. Novel molecules can be generated by sampling latent vectors from the distribution and decoding them with the decoder [20,30]. For instance, the JT-VAE method interpolates reference drugs within the pre-trained VAE’s latent space [20], while GF-VAE randomly samples the space surrounding reference drug molecules in the latent space [31]. However, these approaches have struggled to produce molecules that exhibit both diversity and desired properties.

**GANs.** Generative adversarial networks (GANs) have become widely used in various fields, including image, audio, and video processing [32]. A GAN comprises two parts: a generator *G* and a discriminator *D*. During training, the GAN plays a max-min game, in which *D* learns to differentiate between real and generated data from *G*, while *G* learns to generate more realistic data to deceive *D*. Ultimately, the trained *G* generates realistic data, and the trained *D* improves its ability to classify fake data. In recent years, there has been a surge of GAN-based models applied to molecule design [23,33,34,35]. For example, Mol-GAN trains a GAN from scratch using a large dataset of drug molecules with desired properties [23]. Mol-CycleGAN, on the other hand, trains GANs based on the latent space of pre-trained VAEs [35]. However, these methods require extensive collections of drug molecules for training.

To summarize, none of the previous methods have addressed the challenge of generating a diverse set of desired drugs with only a few references. Few-shot generation has been extensively studied in computer vision [36,37,38]; however, few-shot drug molecule generation remains an ongoing area [39]. Low-data drug discovery techniques, such as few-shot property prediction [40], cannot be directly applied to molecule generation.

## 3. Our Method

**Figure 1:**
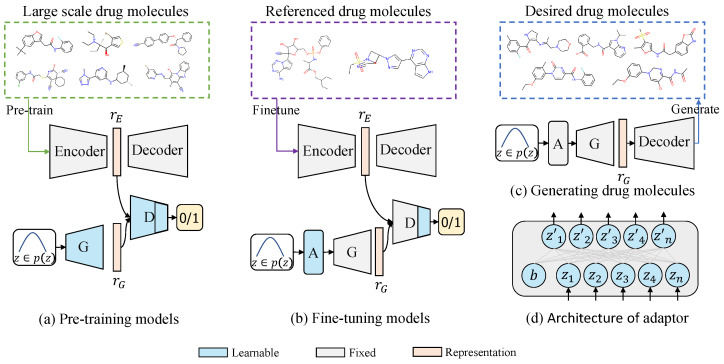
An illustrative diagram of Mol-GenDA. (**a**) The GAN is pre-trained on a large-scale drug molecule dataset. (**b**) For a specific task, the pre-trained GAN is fine-tuned with related drug molecules using a molecule adaptor. (**c**) The fine-tuned GAN is utilized to generate desired drug molecules. (**d**) The architecture of molecule adaptor, where a two-layer neural network is adopted to adapt the original distribution to that of desired drug molecules. A, G, and D denote the molecule adaptor, generator, and discriminator, respectively. Best viewed in color.

To tackle the issue of low-data drug molecule generation, we propose a novel generative domain adaptation approach called Mol-GenDA, inspired by recent work in computer vision [38]. Figure 1 depicts the overall workflow of Mol-GenDA. Firstly, the GAN is pre-trained on a large-scale drug molecule dataset. Then, it is fine-tuned with few-shot reference drug molecules using a lightweight molecule adaptor. Finally, the model is used to generate desired drug molecules.

### 3.1. Large-Scale Pre-Training

We adopted the Junction Tree Variational Autoencoder (JT-VAE) to encode drug molecules into a latent space and decode latent vectors back to drug molecules, as in previous studies on drug molecule generation [23,35,41]. The JT-VAE approach is VAE-based and operates on the graph structure representation of molecules, employing a junction-tree scaffold of molecule sub-components and a graph-structure representation of molecules. Compared to other VAE-based methods that operate on the SMILES representation of molecules, JT-VAE exhibits superior performance, with 100% validity of the generated molecules [30,31]. Pre-training the JT-VAE on a large-scale molecule dataset can improve its representation learning capability. In this work, we take the pre-trained JT-VAE (https://github.com/wengong-jin/icml18-jtnn, accessed on 25 April 2021) on ZINC-250K from previous work [20]. During pre-training and fine tuning, we froze the parameters of JT-VAE.

In this stage, we pre-train the GAN on a large-scale molecule dataset and freeze the JT-VAE. As shown in Figure 1a, the generator *G* produces fake molecule latent vectors, while the VAE encoder produces realistic ones. The discriminator *D* is trained to classify whether the vectors are realistic or generated by *G*. The training strategy is a max-min game, and the objective between the generator *G* and discriminator *D* can be formulated as:

(1) $$\min_{G}\max_{D}\underset{r_{E}∼P_{r}}{E}\left[\log\left(D\left(r_{E}\right)\right)\right]+\underset{r_{G}∼P_{g}}{E}\left[\log\left(1-D\left(r_{G}\right)\right)\right],$$

where $P_{r}$ is the data distribution, $P_{g}$ is the model distribution defined implicitly by $r_{G}=G\left(z\right)$, and z∼p(z) is sampled from a simple noise distribution (a Gaussian distribution is chosen in this work). Following WGAN-GP [42], we introduce the gradient penalty to the model, and the loss function is finally improved as follows:

(2) $$\begin{array}{c} L=\underset{r_{G}∼P_{g}}{E}\left[D\left(r_{G}\right)\right]-\underset{r_{E}∼P_{r}}{E}\left[D\left(r_{E}\right)\right]+\lambda \underset{{\hat{r}}_{E}∼P_{{\hat{r}}_{E}}}{E}\left[{\left({∥\nabla_{{\hat{r}}_{E}}D\left({\hat{r}}_{E}\right)∥}_{2}-1\right)}^{2}\right], \end{array}$$

where ${\hat{r}}_{E}∼P_{{\hat{r}}_{E}}$ is uniformly sampled along the straight lines between pairs of points sampled from the data distribution $P_{r_{E}}$ and the generator distribution $P_{g}$. Algorithm 1 summarizes the details of the pre-training process.
**Algorithm 1:** Large-scale pre-training
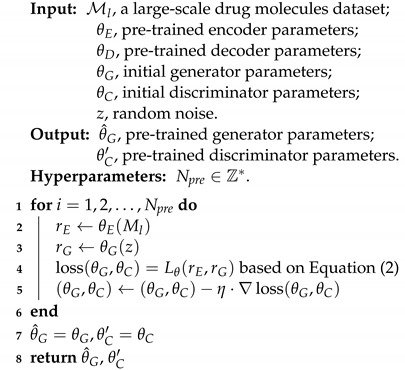


### 3.2. Generative Domain Adaptation

The pre-trained generator of Mol-GenDA has already acquired the ability to generate diverse and high-quality drug molecules, learned from a large-scale drug molecule dataset. The random noise *z* input into the generator of GAN can be viewed as the features used to generate latent drug molecule vectors in the latent space of a pre-trained JT-VAE [43]. These latent vectors can then be decoded by the decoder of JT-VAE to produce drug molecules. The goal of Mol-GenDA fine tuning is to learn the ability to select appropriate features, i.e., the noise *z*, for generating desired drug molecules. However, fine-tuning all the parameters of the generator on a few reference drug molecules carries the risk of overfitting. To address these concerns, we designed a lightweight module called molecule adaptor. As illustrated in Figure 1b, during the fine-tuning process, we freeze the parameters of the generator and only update those of the molecule adaptor. The goal is to transfer the noise distribution, which serves as the input features of the generator to produce latent vectors, to the distribution of reference drug molecules. The architecture of the molecule adaptor is illustrated in Figure 1d, and it can be defined as:

(3) $$z^{\prime }=Az+b$$

where *A* and *b* are the linear projection matrix that controls the variation scale of the latent vector, and a bias vector in the affine module learned in the fine-tuning.

Regarding the discriminator, the first several layers are responsible for feature extraction, while the latter layers perform classification [44]. As the discriminator is a binary classifier, we freeze the first several layers and train only the last *n* layers, aiming to maintain the discriminator’s ability to extract key features while training it to classify whether the drug is desired or not. In this work, we update the last two layers of the discriminator during fine-tuning. Reference drug molecules are fed into the joint model, and the objective is the same as in pre-training, as shown in Equation (2). Algorithm 2 summarizes the details of the generative domain adaptation process.
**Algorithm 2:** Generative Domain Adaptation Fine Tuning
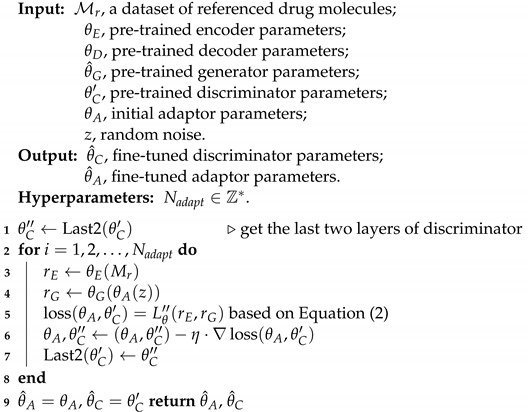


### 3.3. Constrained Molecule Generation

Structure- and property-constrained molecule generation are two common tasks in drug design, and Mol-GenDA can solve their low-data problem.

**Structure-constrained generation.** To achieve structure-constrained generation, we fine-tune the molecule adaptor ${\hat{\theta }}_{A}$ on few-shot molecules with specific structures. With the additional pre-trained generator ${\hat{\theta }}_{G}$ in GAN and decoder ${\hat{\theta }}_{D}$ in VAE, the model generates molecules with desired structures as follows:

(4) $$M_{gen}={\hat{\theta }}_{D}\left({\hat{\theta }}_{G}\left({\hat{\theta }}_{A}\left(z\right)\right)\right),$$

where *z* is randomly sampled noise. Specifically, to generate desired drug molecules, the molecule adaptor adapts the noise distribution to the desired molecule distribution. Then, the noise is input into the generator to obtain the latent vectors $r_{G}$, which can be decoded to the desired drug molecules $M_{gen}$ using the decoder.

**Property-constrained generation.** Generating molecules with desired properties follow a similar process to structure-constrained generation, with the only difference being that the molecule adaptor is fine-tuned on references with high scores for specific properties. Algorithm 3 summarizes the details of constrained molecule generation.
**Algorithm 3:** Constrained Molecule Generation  **Input**: ${\hat{\theta }}_{A}$, trained adaptor parameters;               ${\hat{\theta }}_{G}$, pre-trained generator parameters;               $\theta_{D}$, pre-trained decoder parameters;               *z*, random noise.  **Output**: $M_{gen}$, generated desired drug molecules;  **Hyperparameters**: $N_{\mathrm{gen}}\in Z^{*}$
**1** $r_{G}\leftarrow {\hat{\theta }}_{G}\left({\hat{\theta }}_{A}\left(z\right)\right)$**2** $M_{gen}\leftarrow {\hat{\theta }}_{D}\left(r_{G}\right)$**3** **return** $M_{gen}$

## 4. Results

### 4.1. Data

The model was pre-trained on the ZINC-250K dataset, which contains 250,000 drug-like molecules extracted from the ZINC database [45]. This dataset is commonly used in similar studies [20,35]. To evaluate the model’s performance on few-shot molecule generation, we tested it on six datasets, including the structure- and property-constrained generation. The statistics of these datasets are presented in Table 1.

**Structure-constrained generation.** For downstream tasks of structure-constrained generation, we constructed four subsets with specific structures. Aromatic rings and halogen moieties are two important structural properties of molecules. The **1 ring**, **2 rings**, and **3 rings** datasets contain molecules with one, two, and three rings, respectively. The **Halogen** dataset contains molecules with halogen groups. Each of these datasets contains four subsets of 5-shot, 10-shot, 50-shot, and 100-shot reference drug molecules. To construct these datasets, we randomly selected 100 molecules from the ZINC-250K dataset for each 100-shot dataset. Then, we selected 50 molecules from the 100-shot subsets to construct the 50-shot subsets. The 10-shot subsets were randomly selected from the 50-shot subsets, and the 5-shot subsets were selected from the 10-shot ones. They were constructed to evaluate the model’s ability to perform structural transformations. For instance, the model can execute simple structural modifications such as changing the presence of halogen groups or altering the number of aromatic rings.

**Property-constrained generation. Plogp** and **QED** datasets consist of molecules with top penalized logP (Plogp) and quantitative estimate of drug-likeness (QED) scores, respectively. These datasets evaluate the performance of property-constrained generation with few-shot references. Specifically, we extracted the molecules with the first 5, 10, 50, and 100 top scores of QED and Plogp from the ZINC-250K dataset to create the 5-shot, 10-shot, 50-shot, and 100-shot datasets.

### 4.2. Model and Training Configurations

In our evaluation, the generator *G* of GAN consists of a seven-layer multilayer perceptron (MLP) with 100, 128, 256, 256, 512, 256, and 56 neurons, respectively. The discriminator *D* of GAN contains a 5-layer MLP with 56, 128, 256, 128, and 1 neurons, respectively. The activation functions in the generator and discriminator are *Tanh()* and *LeakyReLU()*, respectively. The adaptor is composed of two-layer MLPs with 57 and 56 neurons, respectively. The architecture of an adaptor is straightforwardly designed according to the length of the latent vector. We pre-trained the GANs for 200 epochs with a mini-batch size of 128 and optimized the objective using the Adam optimizer [46] with a learning rate of 1e-3. The loss was calculated using Equation (2). During the fine-tuning process, we also used the Adam optimizer and fine-tuned the model for 40 epochs with a mini-batch size of 1. For each experiment, we generated 1,000 drug molecules for evaluation. All experiments were conducted on a computing cluster with eight NVIDIA^®^ GeForce^®^ RTX 2080 Ti 11GB GPUs and an Intel^®^ Xeon^®^ Gold 6139 CPU @ 2.30GHz. PyTorch [47] was applied to complete our model and RDKit [48] was used to draw the pictures and estimate the properties of molecules.

### 4.3. Comparison to Previous Methods

**Structure-constrained generation.** We compare our proposed method with GAN and pre-trained GAN, for structure-constrained generation. **GAN** is directly trained with few-shot drug molecule references with specific structures in the latent space of VAE. In this work, we adopted WGAN-GP [42]. On the other hand, **pre-trained GAN** is pre-trained on the ZINC-250K dataset from scratch.

**Property-constrained generation.** In addition to GAN and pre-trained GAN, we compare our proposed method with previous approaches that use reference drugs, including interpolating [31] and random sampling [30] for property-constrained generation. In **random sampling**, the reference drugs are encoded into the latent space using the VAE encoder to obtain their representations. Then, the spaces around these points are randomly sampled with radii of 0.5, 1, and 2, respectively, as used in this work. On the other hand, in **interpolation**, the desired drugs in the latent space are obtained by interpolating between each pair of reference drug latent vectors.

### 4.4. Evaluation Metrics

**Structure-constrained generation.** We evaluate the performance of structure-constrained generation based on diversity, uniqueness, and quality. **Diversity** measures the diversity of generated molecules and is defined as:

(5) $$\begin{array}{c} \mathrm{Diversity}=1-\frac{1}{\left|M_{gen}\right|\left(\left|M_{gen}\right|-1\right)}\sum_{m_{1},m_{2}\in M_{gen}}^{m_{1}\neq m_{2}}sim\left(m_{1},m_{2}\right), \end{array}$$

where $\left|\cdot \right|$, sim(·), and $M_{gen}$ denote the operation of obtaining the number, a similarity calculation method, and the generated molecules, respectively. In this work, we adopt the Tanimoto similarity between two extended-connectivity fingerprint bit vectors. **Uniqueness** measures the degree of variety during sampling and is defined as the ratio between the number of unique samples and valid samples:

(6) $$\mathrm{Uniqueness}=\frac{\left|M_{unique}\right|}{\left|M_{gen}\right|},$$

where $M_{unique}$ denotes the set of unique drugs (i.e., removing duplicated drugs in the generated set). **Quality** is the ratio between the numbers of drug molecules with desired structures and the generated drug molecules, defined as follows:

(7) $$\mathrm{Quality}=\frac{\left|M_{desired}\right|}{\left|M_{gen}\right|},$$

where $M_{desired}$ is the desired drug molecule sets without duplicated molecules.

**Property-constrained generation.** In addition to diversity and uniqueness, we evaluate the performance of property-constrained generation based on the scores of desired properties, including penalized logP(**PlogP**) and quantitative estimate of drug-likeness(**QED**). PlogP is a commonly used property to evaluate molecule optimization models’ performance, as it is relevant in the drug design process. It is defined as the logarithm of the ratio of the concentrations of a solute in two solvents, and it provides a measure of lipophilicity. QED score is another critical metric for drug design, which measures the similarity between a compound’s properties and those of known drugs. QED stands for quantitative estimate of drug-likeness, and it is a widely used measure in drug discovery.

### 4.5. Performance and Discussion

#### 4.5.1. Structure-Constrained Generation

**Figure 2:**
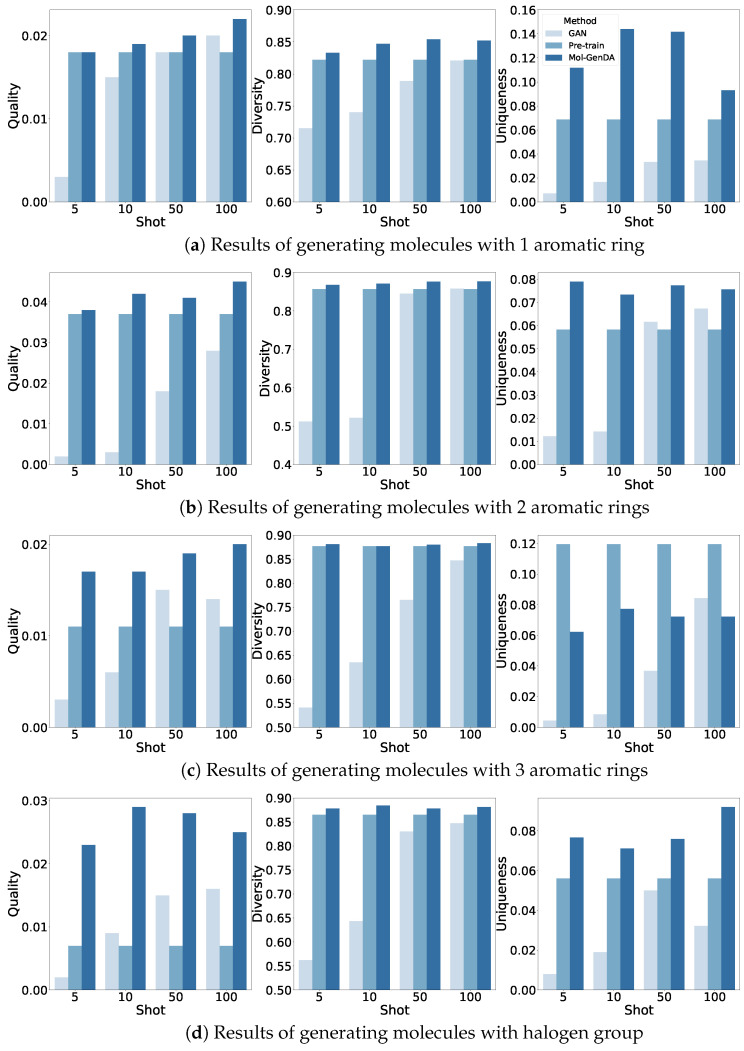
Results of structure-constrained molecule generation.

The results of generating molecules with one, two, and three aromatic rings, as well as halogens, are presented in Figure 2. Overall, Mol-GenDA demonstrates superior performance compared to GAN and pre-trained GAN in terms of diversity, quality, and uniqueness in most cases. While Mol-GenDA performs slightly worse in terms of the uniqueness of generated molecules with three rings, the experimental results still demonstrate its ability to enhance the quality of generation while maintaining diversity.

Specifically, GAN trained from scratch on few-shot reference drug molecules performs worse than both Mol-GenDA and pre-trained GAN, as training a GAN with just a few molecules is challenging. Although the quality and diversity of molecules generated by GAN increase with more reference molecules for training, it is still not enough to train a proper GAN with just 100 reference drug molecules. Pre-trained GAN generates more diversified drug molecules than GAN because GAN is trained with only a few reference drug molecules, which limits its learning space. Additionally, the training data for GAN are only a subset of those of pre-trained GAN, further narrowing down its learning space.

Mol-GenDA outperforms GAN in terms of diversity because the pre-trained generator maintains the knowledge learned from large-scale training drug molecules in pre-training. The diversity of drug molecules generated by pre-trained GAN and Mol-GenDA is similar because both learned from large-scale training drug molecules. Moreover, the diversity of molecules generated by Mol-GenDA varies in a small range since the diversity learned from large-scale pre-training is maintained by freezing the parameters of the generator in the domain adaptation fine tuning.

Mol-GenDA generates more desired drug molecules than pre-trained GAN because of the additional fine-tuning stage that adapts the pre-trained GAN to a specific domain. Training a GAN from scratch is hard because the latent space for the GAN to explore is too large while only a few reference drug molecules are available, making it difficult to learn the common features of the reference drug molecules. In contrast, only part of Mol-GenDA’s parameters are updated in fine tuning, allowing for generating drug molecules with higher quality than GAN and pre-trained GAN.

**Figure 3:**
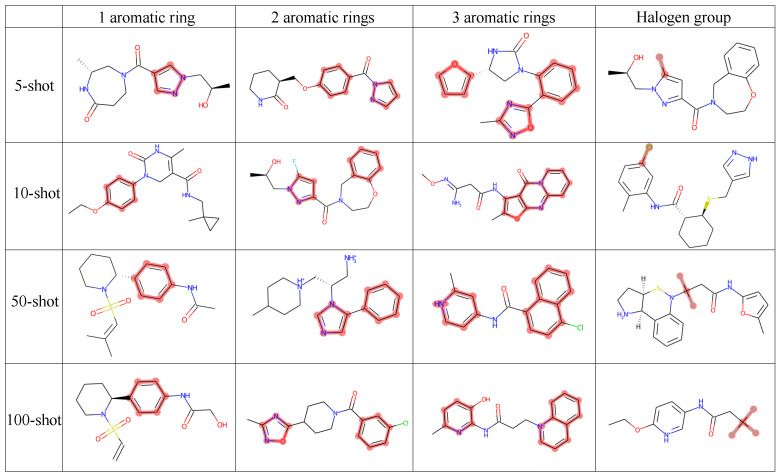
Structure-constrained generation examples of Mol-GenDA. Each row represents the structures that are desired and used for Mol-GenDA training, including 1, 2, and 3 aromatic rings, and the halogen group. Each column corresponds to the generation with 5-shot, 10-shot, 50-shot, and 100-shot reference drug molecules, respectively. The red highlight indicates the corresponding structures, i.e., the aromatic ring and halogen group. All molecules shown in the figure are randomly selected from the corresponding task’s generation.

Figure 3 shows the generation examples of Mol-GenDA, with molecules randomly selected from the generation of each task. We can see that Mol-GenDA successfully generates molecules with desired structures.

#### 4.5.2. Property-Constrained Generation

The QED- and PlogP-constrained drug molecule generation results are presented in Table 2 and Table 3, respectively. Mol-GenDA outperforms other methods in terms of QED and PlogP scores, demonstrating its capability for few-shot property-constrained molecule generation.

Interpolation and random sampling methods generate drug molecules with similar representation vectors in the latent space, and increasing the radius of random sampling improves the diversity of generated molecules by ensuring more diverse representation vectors. However, learning the target property features from few-shot reference drug molecules is challenging. Pre-trained GANs are trained on large-scale drug molecules without accessing the specific property features of the target molecules, which limits their ability to generate desired molecules. Similarly, GANs trained from scratch also struggle to capture the target features necessary for generating the desired molecules, although their performance can be improved with the aid of a well-trained VAE.

Mol-GenDA outperforms other baseline methods in terms of property score, but it performs worse in diversity than interpolation and random sampling, and better than GANs and pre-trained GANs. Since the representation vectors generated by interpolation and random sampling are different naturally, the diversity of the molecules decoded from these vectors is guaranteed, but they fail to generate molecule drugs with desired properties. Overall, Mol-GenDA appropriately addresses the challenge of generating desired drug molecules with few-shot references.

**Figure 4:**
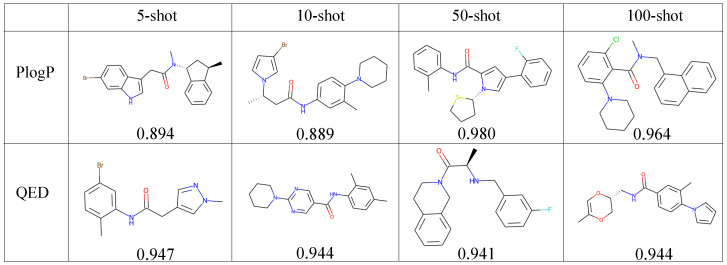
Property-constrained generation examples of Mol-GenDA. Each row represents the generation using 5-shot, 10-shot, 50-shot, and 100-shot reference drug molecules, respectively. Each column indicates the desired properties used for Mol-GenDA training, including QED and PlogP. The selected molecules in each row have the highest property scores for their corresponding task. The property scores are below each molecule.

Figure 4 displays the generated candidate molecules with the highest property scores for their respective tasks. We can see that Mol-GenDA successfully generates molecules with higher scores of desired properties.

#### 4.5.3. Activity: Dopamine Receptor D2

**Figure 5:**
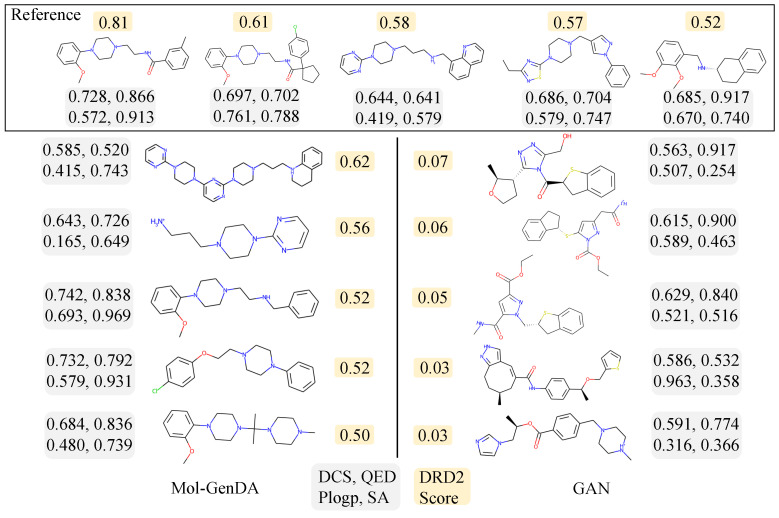
The 5 reference drugs and candidates generated by Mol-GenDA and GAN for DRD2. The numbers beside each molecule represent the drug candidate scores (DCS), QED, PlogP, SA, respectively, with higher scores indicating better generation results. DRD2 is the probability that one molecule can trigger the biological activity of DRD2. The drug molecules are arranged in order of DRD2 Score.

To further validate the effectiveness of our Mol-GenDA, we conduct experiments on generating bioactive drug molecules with Dopamine Receptor D2 (DRD2) as the biological target. DRD2 score is the probability that one molecule can trigger the biological activity of DRD2. Specifically, we utilize a machine learning-based score model from [35] to select the top five reference molecules that can trigger the biological activity of DRD2. Mol-GenDA then generates desired molecules with the five reference molecules. Figure 5 shows the top five molecules generated by Mol-GenDA and GAN trained from scratch in terms of DRD2 score. We can see that the molecules generated by Mol-GenDA share similar substructures with reference molecules and maintain the diversity score of 0.921, which is higher than that of GAN (i.e., 0.770). Furthermore, Mol-GenDA generates drug molecules with much higher DRD2 scores (i.e., the average DRD2 score of the top five molecules is 0.544) than those of GAN (i.e., the average DRD2 score is 0.048). Mol-GenDA outperforms GAN trained from scratch on both DRD2 score and diversity because of the fine-tuning paradigm in Mol-GenDA, which generates molecules with desired properties and maintains diversity. The pre-training enables Mol-GenDA to generate valid molecules, and the fine-tuning paradigm introduces an adaptor to learn the distribution of drug molecules with desired properties while freezing the parameters of the generator, which maintains the diversity of generated drug molecules. Additionally, during the fine-tuning process, only the last two layers of the discriminator are trained, which simplifies the training procedure. However, for GAN trained from scratch, exploring the enormous latent space for drug molecules with desired properties is difficult. Furthermore, we find that the QED and PlogP scores of Mol-GenDA (i.e., 0.724 and 0.491) are comparable to those of GAN (i.e., 0.756 and 0.503). Similarly, the two methods also achieved comparable performance on the other two properties, drug candidate score (DCS) (i.e., 0.595 and 0.609) and synthetic accessibility (SA) (i.e., 0.531 and 0.503). This is because both of them contain the VAE module which is taken from previous works [20] and pre-trained to be full of valid drug molecules in the latent space for both methods.

### 4.6. Case Study: Drug Generation for COVID-19

**Figure 6:**
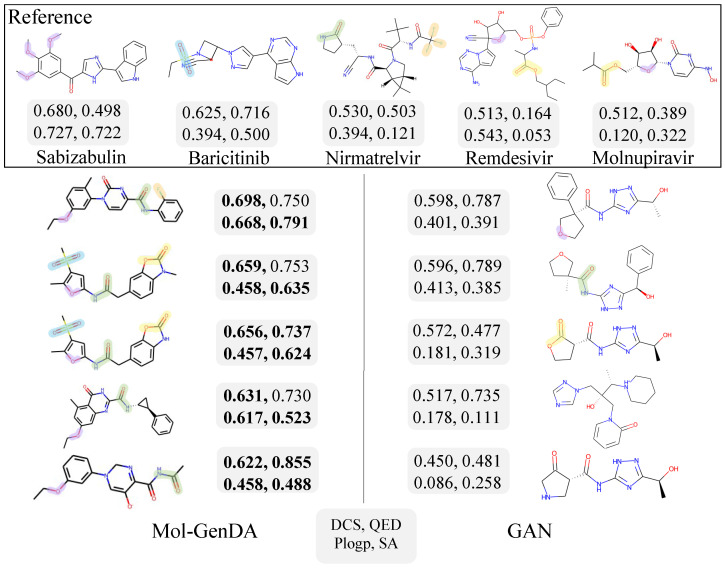
The 5 reference drugs and candidates generated by GAN and Mol-GenDA for COVID-19. The numbers beside each molecule represent the drug candidate scores (DCS), QED, PlogP, SA, respectively, with higher scores indicating better generation results. The highlight with the same color indicate the same substructure. The drug molecules are arranged in order of drug candidate score.

Due to the sudden outbreak of COVID-19 and the limited availability of drugs for similar diseases, we utilized Mol-GenDA to identify effective candidate drugs for COVID-19. We collected five established drugs known to be useful in treating COVID-19 as reference drugs to fine-tune the pre-trained GAN in Mole-GenDA, including Remdesivir [49], Nirmatrelvir [50], Baricitinib [51], Sabizabulin [52], and Molnupiravir [53]. Figure 6 shows the candidate drugs generated by the GAN trained from scratch and our Mol-GenDA, which are all valid and novel. The drugs generated by Mol-GenDA maintain both diversity and similarities to the reference drugs, achieving a diversity score of 0.870. The QED and PlogP scores of the generated drugs are 0.765 and 0.532, respectively, and the average synthetic accessibility (SA) score for our drug candidates is 0.612. In comparison, the diversity, QED score, PlogP score, and SA score of the drugs generated by the GAN trained from scratch are 0.747, 0.654, 0.252, and 0.293, respectively, with only the QED score being comparable to Mol-GenDA.

## 5. Conclusions

**Advantages.** In this study, we proposed Mol-GenDA, a molecule generative domain adaptation approach for low-data drug discovery, which addressed the challenge of generating drugs with both diversity and quality using only a few reference drugs. We introduced a lightweight molecule adaptor that efficiently adapts the pre-trained generator to the target disease domain with a few reference drug molecules. We first pre-trained the GAN on ZINC-250K, a large-scale drug-like dataset, then froze the parameters of the pre-trained generator and optimized only the molecule adaptor during fine tuning on the new disease dataset. This approach makes the generator leverage the prior knowledge learned in the source domain to improve the generation quality and diversity in the target domain. Extensive experimental results on both structure-constrained and property-constrained generation consistently demonstrated the superiority of Mol-GenDA over previous works in terms of common evaluation metrics on the low-data drug design task.

**Limitations.** In the few-shot generation setting, Mol-GenDA outperformed previous works in structure-constrained generation and most methods in property-constrained generation. However, its diversity in generating molecules with desired properties is not as good as that of interpolation and random sampling methods. This is due to the limited space explored by Mol-GenDA during fine-tuning, as only the adaptor and the last two layers of the discriminator are updated, resulting in a trade-off between diversity and desired properties.

Although Mol-GenDA still requires a few reference drugs, our future work will focus on generating desired drugs with one-shot learning or directly controlling the structures of generated drugs. Specifically, we aim to generate desired drug molecules using a large chemical language model, utilizing only the description of the molecule properties or structures to retrieve the knowledge from the large chemical language model.

## Figures and Tables

**Table 1:** Statistics of the datasets, where Num. and Diver. indicate number and diversity, respectively.

Task	Dataset	Num.	Diver.	Plogp	QED	1 Ring	2 Rings	3 Rings	Halogen
Pre-training	ZINC	2.5 × 10${}^{5}$	0.915	0.561	0.731	75,580	98,222	47,603	87,556
Structure- constrained	1 ring	5	0.902	0.519	0.799	5	-	-	-
		10	0.905	-	-	10	-	-	-
		50	0.906	-	-	50	-	-	-
		100	0.909	-	-	100	-	-	-
	2 rings	5	0.893	0.668	0.839	-	5	-	-
		10	0.905	-	-	-	10	-	-
		50	0.906	-	-	-	50	-	-
		100	0.908	-	-	-	100	-	-
	3 rings	5	0.889	-	-	-	-	5	-
		10	0.902	-	-	-	-	10	-
		50	0.907	-	-	-	-	50	-
		100	0.908	-	-	-	-	100	-
	Halogen	5	0.895	-	-	1	-	3	5
		10	0.900	-	-	2	3	3	10
		50	0.908	-	-	11	28	6	50
		100	0.911	-	-	25	52	15	100
Property- constrained	QED	5	0.864	0.604	0.947	-	-	-	-
		10	0.888	0.599	0.947	-	-	-	-
		50	0.891	0.602	0.947	-	-	-	-
		100	0.893	0.602	0.947	-	-	-	-
	PlogP	5	0.878	1.000	0.292	-	-	-	-
		10	0.891	1.000	0.288	-	-	-	-
		50	0.901	1.000	0.357	-	-	-	-
		100	0.905	1.000	0.390	-	-	-	-

**Table 2:** Results of QED-constrained generation.

		Inter- Polate	Random Sampling			GAN	Pre-Train GAN	Mol- GenDA
			0.5	1	2			
5-shot	QED	0.681	0.486	0.519	0.497	0.729	0.749	0.771
	Diversity	0.868	0.908	0.923	0.930	0.850	0.866	0.859
	Uniqueness	0.129	0.929	0.979	0.994	0.094	0.243	0.240
10-shot	QED	0.738	0.439	0.525	0.516	0.753	0.749	0.769
	Diversity	0.886	0.902	0.920	0.927	0.832	0.866	0.865
	Uniqueness	0.395	0.929	0.979	0.994	0.900	0.243	0.246
50-shot	QED	0.721	0.439	0.506	0.501	0.736	0.749	0.749
	Diversity	0.897	0.910	0.922	0.928	0.841	0.866	0.862
	Uniqueness	0.998	0.840	0.997	0.996	0.073	0.242	0.243
100-shot	QED	0.722	0.467	0.521	0.683	0.748	0.749	0.762
	Diversity	0.897	0.914	0.921	0.927	0.852	0.866	0.866
	Uniqueness	0.988	0.897	0.999	0.997	0.066	0.243	0.263

**Table 3:** Results of PlogP-constrained generation.

		Inter- Polate	Random Sampling			GAN	Pre-Train GAN	Mol- GenDA
			0.5	1	2			
5-shot	Plogp	0.651	0.639	0.547	0.519	0.519	0.568	0.682
	Diversity	0.880	0.908	0.923	0.930	0.825	0.866	0.936
	Uniqueness	0.234	0.929	0.979	0.994	0.069	0.243	0.252
10-shot	Plogp	0.660	0.669	0.667	0.609	0.562	0.568	0.679
	Diversity	0.899	0.871	0.920	0.927	0.837	0.866	0.866
	Uniqueness	0.612	0.827	0.994	0.995	0.065	0.243	0.255
50-shot	Plogp	0.635	0.617	0.614	0.594	0.547	0.568	0.677
	Diversity	0.893	0.910	0.922	0.928	0.849	0.866	0.865
	Uniqueness	0.982	0.840	0.997	0.996	0.068	0.243	0.232
100-shot	Plogp	0.659	0.653	0.607	0.590	0.484	0.568	0.672
	Diversity	0.913	0.914	0.921	0.928	0.864	0.866	0.865
	Uniqueness	0.985	0.897	0.999	0.994	0.085	0.243	0.236

## Data Availability

The datasets employed in our study can be found at Google Drive: https://drive.google.com/drive/folders/10Z2n6co40abDIkuNlrNgDZkO1e7K-g91?usp=sharing, accessed on 11 January 2022. All code has been deposited and is publicly available on Github: https://github.com/zjuKeLiu/Mol-GenDA, accessed on 3 May 2023.
